# Developmental Programming of Cardiovascular Dysfunction by Prenatal Hypoxia and Oxidative Stress

**DOI:** 10.1371/journal.pone.0031017

**Published:** 2012-02-13

**Authors:** Dino A. Giussani, Emily J. Camm, Youguo Niu, Hans G. Richter, Carlos E. Blanco, Rachel Gottschalk, E. Zachary Blake, Katy A. Horder, Avnesh S. Thakor, Jeremy A. Hansell, Andrew D. Kane, F. B. Peter Wooding, Christine M. Cross, Emilio A. Herrera

**Affiliations:** Department of Physiology, Development and Neuroscience, University of Cambridge, Cambridge, United Kingdom; University of Las Palmas de Gran Canaria, Spain

## Abstract

Fetal hypoxia is a common complication of pregnancy. It has been shown to programme cardiac and endothelial dysfunction in the offspring in adult life. However, the mechanisms via which this occurs remain elusive, precluding the identification of potential therapy. Using an integrative approach at the isolated organ, cellular and molecular levels, we tested the hypothesis that oxidative stress in the fetal heart and vasculature underlies the molecular basis via which prenatal hypoxia programmes cardiovascular dysfunction in later life. In a longitudinal study, the effects of maternal treatment of hypoxic (13% O_2_) pregnancy with an antioxidant on the cardiovascular system of the offspring at the end of gestation and at adulthood were studied. On day 6 of pregnancy, rats (n = 20 per group) were exposed to normoxia or hypoxia ± vitamin C. At gestational day 20, tissues were collected from 1 male fetus per litter per group (n = 10). The remaining 10 litters per group were allowed to deliver. At 4 months, tissues from 1 male adult offspring per litter per group were either perfusion fixed, frozen, or dissected for isolated organ preparations. In the fetus, hypoxic pregnancy promoted aortic thickening with enhanced nitrotyrosine staining and an increase in cardiac HSP70 expression. By adulthood, offspring of hypoxic pregnancy had markedly impaired NO-dependent relaxation in femoral resistance arteries, and increased myocardial contractility with sympathetic dominance. Maternal vitamin C prevented these effects in fetal and adult offspring of hypoxic pregnancy. The data offer insight to mechanism and thereby possible targets for intervention against developmental origins of cardiac and peripheral vascular dysfunction in offspring of risky pregnancy.

## Introduction

Cardiovascular disease is the greatest killer in the world today, imposing a substantial burden on every nation's health and wealth [1]. The concept that environmental risk factors, such as smoking and obesity, interact with our genetic makeup to determine susceptibility to cardiovascular dysfunction is well accepted [2]. However, only comparatively recently, it has become apparent that the quality of the prenatal environment may also play a role [3], [4]. In pregnancy complicated with adverse intrauterine conditions, adaptations are enforced in the unborn child and placenta, which can alter the development of key organs and systems, such as the heart and circulation. Whilst they are necessary to maintain viable pregnancy and sustain life before birth, these adaptations come at a cost, triggering many biological trade-offs for later life. Early insults at critical stages of development may therefore lead to permanent changes in tissue structure and function, a concept now known as programming [5]. The concept creates an exciting window of opportunity to halt the development of cardiac and vascular dysfunction at its very origin, bringing preventive medicine back into the womb. However, the mechanisms underlying developmental programming remain elusive, precluding the identification of potential avenues for clinical therapy [6], [7].

One of the most common adverse conditions in complicated pregnancy is a reduction in oxygen delivery to the developing young. The fetal defence to a short-term episode of hypoxia includes the redistribution of blood flow away from peripheral circulations towards essential vascular beds, such as those perfusing the brain [8]. This brain-sparing effect is conserved across all species studied from the reptilian and avian embryo to the mammalian fetus, including the sheep, non-human and human primate [9]. Should the duration of the hypoxic challenge become prolonged, the initial homeostatic cardiovascular defences persist [10], [11]. In response to chronic hypoxia, sustained redistribution of blood flow towards essential circulations ensures fetal survival, but the adaptation claims a number of unwanted side-effects. The best described is asymmetric fetal growth restriction [11]. More recently, it has also been reported that chronic hypoxia during pregnancy promotes an increase in fetal cardiac afterload, imposing a strain on the developing heart and major vessels, leading to ventricular and aortic wall thickening [12]–[14]. Severe hypoxia from early development may overpower ventricular compensatory mechanisms, switching the cardiac phenotype to one of myocardial thinning [15], [16]. Other elegant experiments have shown that prenatal hypoxia can alter cardiac performance and increase its susceptibility to ischaemia-reperfusion injury, in addition to promoting endothelial dysfunction in adult life [16]–[26]. Thus, intrauterine hypoxia is not only an immediate threat to fetal life, but is also provides a strong stimulus for a developmental origin of heart and vascular disease. However, the mechanisms through which prenatal hypoxia programmes cardiac and endothelial dysfunction in adulthood have not been identified. Therefore, no study to date has been able to prevent the programming of cardiac and vascular dysfunction in adulthood by prenatal hypoxia.

Hypoxia is a potent stimulus for the generation of reactive oxygen species (ROS) [27]. Under physiological conditions, ROS are important mediators of a wide variety of cell functions through their actions on redox-sensitive transcription factors. However, excessive generation of ROS and/or a fall in antioxidant defences can lead to indiscriminate damage, resulting in cellular oxidative stress [27]. Here, we tested the hypothesis that oxidative stress in the fetal heart and vasculature underlies the molecular basis through which prenatal hypoxia contributes to the developmental programming of cardiac and endothelial dysfunction. The hypothesis was tested using an integrative approach at the isolated organ, cellular and molecular levels, in a longitudinal study in rats. We investigated the effects of treatment of maternal hypoxic pregnancies with an antioxidant on the cardiovascular system of the offspring at two stages of life: in the fetal period at the end of gestation and at 4 months of adult age.

## Methods

### Ethics Statement

Experiments were approved by the Ethical Review Committee of the University of Cambridge and were carried out under the UK Animals (Scientific Procedures) Act 1986.

Wistar rat pregnancies were established as described [13], [28]. On day 6 of pregnancy, rats were randomly divided into 4 groups (n = 20 per group): control and hypoxic pregnancy, with and without vitamin C treatment (5 mg.ml^−1^ maternal drinking water freshly prepared every day). Pregnant rats subjected to hypoxia were placed inside a chamber, which combined a PVC isolator with a nitrogen generator [13], [28]. Pregnancies undergoing hypoxia were maintained at a constant inspired fraction of oxygen of 13% from day 6 to 20 of gestation (term is *ca.* 21 days). At day 20 of gestation, one set of dams (n = 10) from each group was anesthetised with isoflurane and then maintained by a mixture of ketamine (40 mg•kg^−1^) and xylazine (5 mg•kg^−1^) injected intraperitoneally. A maternal blood sample (1 ml in EDTA plus 0.5 ml in metaphosphoric acid) for measurement of ascorbic acid was taken by cardiac puncture, the pregnant uterus was exposed via a mid-line incision and the anesthetised pups were killed via spinal transection. Dams which had been housed in the hypoxic chamber underwent the procedure while being spontaneously ventilated with 13% O_2_ via a small cone. All fetuses and associated placentas were isolated and weighed. Additional maternal and fetal blood was taken for measurement of haematocrit in duplicate. In all pups, the ano-genital distance was measured with digital callipers for determination of sex. Only cardiovascular tissues associated with one male pup per litter per measured outcome variable were used to control for sex and within-litter variation. Therefore, the fetal thorax (containing heart and aorta) was immersion fixed from one male per litter per group (n = 8) and the fetal heart was frozen from a littermate male per litter per group (n = 8) for subsequent stereological, histological or molecular analyses. The remaining 10 litters per group were allowed to deliver. Following determination of birth weight, litters were culled to 4 males and 4 females to standardise nutritional access and maternal care [13]. At weaning, only male offspring were raised to adulthood. At 4 months, following weighing, 1 male from each litter per outcome variable underwent euthanasia and tissues were either perfusion fixed for stereological and histological analyses (n = 8 per group), or frozen for molecular analysis (n = 8 per group), or dissected for the isolated organ preparations (n = 8 per group).

### Determination of Ascorbic Acid

Reversed-phase high-performance liquid chromatography (HPLC) with electrochemical detection was used to analyse ascorbic acid, based on the method of Iriyama et al. with modifications [29], [30]. Maternal plasma previously acidified 1∶1 with ice-cold 10% metaphosphoric acid (MPA) was centrifuged and the supernatant stored at −70°C. This supernatant was thawed on ice and 50 µl added to 400 µl HPLC grade water, 50 µl of 50% MPA and 200 µl of HPLC grade heptane. The samples were then mixed on a vortex stirrer for 30 s prior to centrifugation at 13,000 r.p.m. for 5 min at 4°C. The lower (aqueous) layer was then removed and transferred to a 0.8 ml HPLC vial. Aliquots of 20 µl were injected onto a 4.6×250 mm, 5 µm C18 Apex II column with guard (Jones Chromatography, Glamorgan, UK) and eluted with a 0.2 mol/l K_2_HPO_4_-H_3_PO_4_ (pH 2.1) mobile phase containing 0.25 mmol/l octane sulfonic acid at a flow rate of 1.0 ml/min. An electrochemical detector (EG & G Instruments, Wokingham, UK) was used for detection, with the working electrode set at 800 mV and a sensitivity 0.2 µamp. Final concentrations for ascorbic acid were calculated with external standards which were run simultaneously. The coefficient of variation of analysis was <5%, with a minimum detection limit of 0.1 µM.

### Stereology, histology and molecular biology

Fetal thoraces and adult hearts and aortas were embedded in paraffin, exhaustively sectioned (10 µm, Leica RM 2235 microtome, Germany) and processed for haematoxylin and eosin (H&E) staining. Fetal aortas were also processed for immuno-reactivity to nitrotyrosine (1∶100, Cayman, California, USA). Nitrotyrosine is a footprint for peroxinitrite generation and an established indicator of vascular oxidative stress [27]. Sections were incubated in primary antibody for 24 h at 4°C, after which they were incubated in secondary antibody with bound gold particles (1∶1000, Jackson Research, Stratech Ltd., UK). Sections were developed using an enhancer (Amersham, UK) and, then cover-slipped.

Quantitative analyses of fixed tissue were performed using an Olympus BX-50 microscope, fitted with a motorised specimen stage and microcator. All analyses were performed using the Computer Assisted Stereology Toolbox (CAST) version 2.0 program (Olympus, Denmark), with the observer blind to the treatment groups. Cardiac and aortic areas were determined using a point grid, which was superimposed on the sections and viewed using a ×10 objective. Points falling on the left ventricular wall plus inter-ventricular septum, left lumen, right ventricle wall and right lumen, or aortic wall or lumen, were counted and the areas were calculated as:
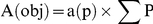



Where A(obj) is the estimated area, a(p) is the area associated with each point and ΣP is the sum of points falling on the relevant area, averaged over the sections.

Western blots were performed using 20 µg aliquots of protein from fetal or adult hearts, resolved on 10% Tris-glycine-SDS polyacrylamide gels (Bio-Rad, Hertfordshire, UK) and immobilised on PVDF membranes. Antibodies to β-actin (1∶50,000; Sigma A5441, USA) or HSP70 (1∶20,000; Stressgen Spa812C, UK), a robust index of cardiac oxidative stress [21], [27], were incubated in 5% milk in 1% Tween-20 (TBS-T, Sigma-Aldrich, UK) overnight at 4°C. Membranes were then incubated for 1 hour in a secondary antibody conjugated to horseradish peroxidase (donkey anti-rabbit IgG or sheep anti-mouse IgG; 1∶10,000, GE Healthcare, UK). Proteins were detected using enhanced chemiluminescence (ECL, Amersham Biosciences, UK), exposed to X-ray film and developed (Fuji FPM100A Processor). Band densities were quantified and expressed relative to β-actin (ImageJ software, NIH).

### In vitro wire myography

Second order femoral arteries (internal diameter in µm: N:269.9±16.0; H: 299.5±20.6; HC: 290.1±20.7; NC:252.7±20.2, P = NS) were mounted on a four-chamber small-vessel wire myograph (Multi Wire Myograph System 610 M, DMT, Denmark) [28], [31]. Relaxant responses to sodium nitroprusside (SNP) and to methacholine (SNP: 10^−10^–10^−4^; MetCh: 10^−10^–10^−6^ mol.L^−1^) were determined after pre-contraction with phenylephrine (PE, 10^−5^ mol.L^−1^). Additional concentration-response curves to MetCh were determined following incubation with either L-NAME (10^−5^ mol.L^−1^) alone or after both L-NAME and indomethacin (10^−6^ mol.L^−1^). Between experiments, vessels were washed repeatedly with Krebs solution and allowed to equilibrate for at least 20 minutes. Concentration-response curves were analysed using an agonist-response best-fit line. The maximal relaxant response (%R_max_) was expressed as percentage of the contraction induced by PE and the vascular sensitivity was expressed as pD_2_ (-logEC_50_). The contribution of NO-dependent mechanisms to the relaxation induced by MetCh was calculated by subtracting the area under the curve (AUC) for MetCh – the AUC for MetCh + LNAME. The contribution of NO-independent mechanisms was calculated by the AUC for MetCh + LNAME [31].

### Langendorff preparation

The same adult male that provided femoral resistance vessels for *in vitro wire myography* provided the heart for the isolated Langendorff preparation, making investigation of cardiac and vascular function in adulthood from the same individual animal possible. Immediately after dissection, isolated hearts were perfused at constant pressure (75 mmHg) under Langendorff mode, with recirculating Krebs-Henseleit bicarbonate solution containing (mM.L^−1^) 120 NaCl, 4.7 KCl, 1.2 MgSO_2_.7H_2_O, 1.2 KH_2_PO_4_, 25 NaHCO_3_, 10 glucose, and 1.3 CaCl_2_.2H_2_O, filtered through a 5 µm cellulose nitrate filter (Millipore, Bedford, MA, USA) and gassed with O_2_:CO_2_ (95∶5) at 37°C. A small flexible non-elastic balloon was inserted into the left ventricle through the left atrium. The balloon was filled with saline and attached to a rigid saline-filled catheter connected to a calibrated pressure transducer (Argon Medical Devices, Texas, USA). The balloon volume was adjusted to 150 µl to obtain left ventricular end diastolic pressure (LVEDP) recording of approximately 5–10 mmHg. After an initial 15-minutes stabilisation period, basal heart rate (HR), left ventricular systolic pressure (LVSP) and LVEDP were recorded. Left ventricular developed pressure (LVDP) was calculated as LVSP-LVEDP. The rate-pressure product (RPP) was calculated as HR×LVSP. The maximum first derivative of the left ventricular pressure (dP/dt_max_) was calculated using an M-PAQ data acquisition system (Maastricht Programmble AcQusition System, Netherlands). Cardiac responsiveness to carbachol (carbamylcholine chloride, Sigma-Aldrich Co. Ltd, Poole, UK) and to isoprenaline ((−)-isoproterenol (+)-bitartrate salt, Sigma-Aldrich Co. Ltd, Poole, UK) was also investigated.

### Data and statistical analyses

The experimental and statistical design was stringent to account for sex differences and within litter variation. Comparisons of variables derived from more than one offspring per litter, such as birth weight, placental weight and placental efficiency were performed using a Generalised Mixed Linear Model Analysis. Other comparisons were of outcome variables derived from only one male offspring per litter per experimental groups. These comparisons were therefore assessed using a One Way ANOVA with the Tukey post hoc test. For all comparisons, significance was accepted when P<0.05.

## Results

**Figure 1:**
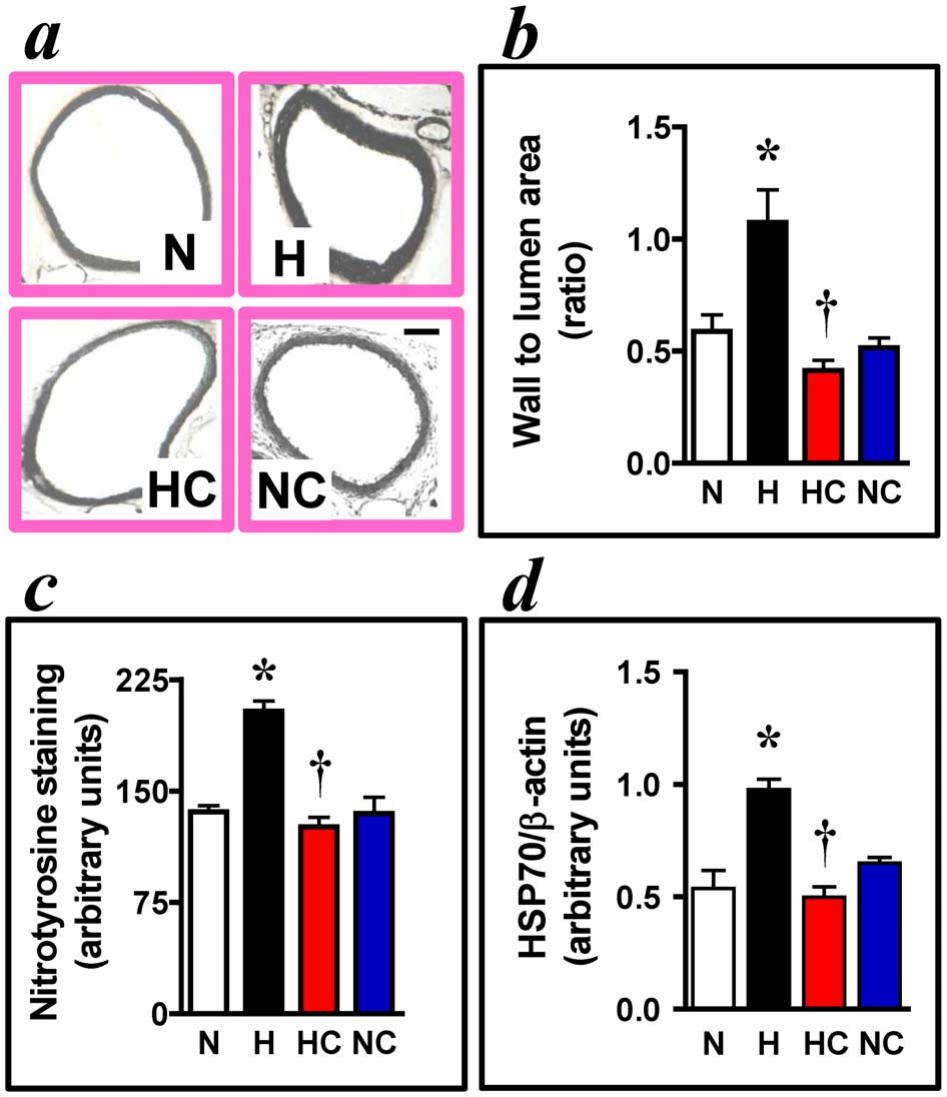
Fetal aorta and heart. Photomicrographs of individual examples of sections of the fetal aorta are shown in *a*. Bar scale is 100 µm. Values are mean±S.E.M. of the wall to lumen area ratio *b*; the density of nitrotyrosine staining in the aortic wall *c*, and the protein expression of HSP70 in the fetal heart *d*. Groups are: Normoxia, n = 8 (N,□), Hypoxia, n = 8 (H,▪), Hypoxia+Vitamin C, n = 8 (HC, red histogram) and Normoxic+Vitamin C, n = 8 (NC, blue histogram). Significant (P<0.05) differences are: * vs. all, † vs. H, One-Way ANOVA with Tukey Test.

In fetal offspring at day 20 of gestation, the aortic wall thickness was significantly enhanced in hypoxic pregnancy (Fig. 1A, 1B). This occurred together with significant elevations in two indices of oxidative stress in the fetal heart and vasculature: cardiac expression of HSP70 and aortic wall nitrotyrosine staining, respectively (Fig.1C, 1D). However, neither the cardiac weight nor the morphology of the left or right ventricle was affected by hypoxic pregnancy (Table 1). Maternal treatment with vitamin C in hypoxic pregnancy restored the aortic wall thickness, aortic wall nitrotyrosine staining and the cardiac expression of HSP70 to values measured in normoxic pregnancy (Fig. 1A–D). In fetal offspring, maternal treatment with vitamin C in normoxic pregnancy did not affect the morphology of the heart or aorta, or the levels of nitrotyrosine in the aortic wall or of HSP70 in the heart (Table 1; Fig. 1A–D).

**Table 1:** Maternal and offspring data.

		N	H	HC	NC
**Mother**	**ascorbic acid**	18.7±2.1	20.0±2.5	31.2±3.0	32.2±2.4*
	(µmol.L^−1^)	(n = 7)	(n = 7)	(n = 7)	(n = 7)
	**haematocrit**	30.2±0.5	35.8±0.2*	36.1±0.4*	30.9±0.7
	(%)	(n = 7)	(n = 7)	(n = 7)	(n = 7)
**Offspring at the end of gestation**	**haematocrit**	34.1±0.2	41.1±0.5*	41.9±0.4*	35.3±0.7
	(%)	(n = 7)	(n = 7)	(n = 7)	(n = 7)
	**birth weight**	6.3±0.6	6.2±0.5	6.6±0.5*	6.8±0.5*
	(g)	(n = 42)	(n = 43)	(n = 35)	(n = 33)
	**placental weight**	0.61±0.01	0.66±0.02*	0.69±0.01*	0.58±0.01
	(g)	(n = 63)	(n = 49)	(n = 60)	(n = 60)
	**litter size**	13.3±0.9	14.0±0.5	12.2±0.9	12.7±0.6
	(n)	(n = 7 dams)	(n = 6 dams)	(n = 6 dams)	(n = 7 dams)
	**absolute heart weight**	25.4±1.7	26.0±1.0	24.4±1.1	22.6±0.5
	(mg)	(n = 18)	(n = 16)	(n = 16)	(n = 18)
	**relative heart weight**	0.70±0.02	0.71±0.02	0.67±0.03	0.67±0.01
	(%)	(n = 18)	(n = 16)	(n = 16)	(n = 18)
	**Left ventricular area**	1.55±0.05	1.49±0.06	1.67±0.07	1.64±0.05
	(mm^2^)	(n = 6)	(n = 6)	(n = 8)	(n = 7)
	**Right ventricular area**	0.99±0.06	0.88±0.08	1.03±0.07	1.01±0.08
	(mm^2^)	(n = 6)	(n = 6)	(n = 8)	(n = 7)
**Offspring at 4 months**	**body weight**	550.9±9.0	540.9±12.6	554.4±8.3	527.6±11.5
	(g)	(n = 25)	(n = 20)	(n = 23)	(n = 23)
	**absolute heart weight**	1.7±0.1	1.6±0.1	1.6±0.1	1.6±0.1
	(g)	(n = 25)	(n = 20)	(n = 23)	(n = 23)
	**relative heart weight**	0.30±0.01	0.30±0.01	0.29±0.01	0.31±0.01
	(%)	(n = 25)	(n = 20)	(n = 23)	(n = 23)
	**Left ventricular area**	27.1±3.2	32.7±1.7	32.7±3.9	28.2±4.4
	(mm^2^)	(n = 5)	(n = 5)	(n = 6)	(n = 6)
	**Right ventricular area**	13.5±1.3	15.8±2.4	13.5±1.3	11.2±1.6
	(mm^2^)	(n = 5)	(n = 5)	(n = 6)	(n = 6)
	**Cardiac HSP70 protein**	0.80±0.09	0.81±0.09	0.78±0.08	0.79±0.05
	**expression** (/β actin)	(n = 8)	(n = 8)	(n = 8)	(n = 8)
	**Aorta wall:lumen area**	0.23±0.02	0.22±0.01	0.23±0.01	0.22±0.01
	ratio	(n = 8)	(n = 8)	(n = 8)	(n = 8)
	**heart rate**	243±20	255±21	235±14	260±14
	(bpm)	(n = 6)	(n = 6)	(n = 7)	(n = 7)
	**LVDP**	84.8±7.9	104.7±9.7	100.0±5.2	92.4±4.8
	(mmHg)	(n = 6)	(n = 6)	(n = 7)	(n = 7)
	**LVEDP**	7.1±1.8	9.8±1.3	8.1±1.6	11.2±2.5
	(mmHg)	(n = 6)	(n = 6)	(n = 7)	(n = 7)

Values are mean±S.E.M. for dams and offspring of Normoxic (N), Hypoxic (H), Hypoxic+Vitamin C (HC) and Normoxic+Vitamin C (NC) pregnancy. Placental weight, fetal haematocrit and fetal heart weight were taken at gestational day 20. Values for heart rate, left ventricular developed pressure (LVDP) and left ventricular end diastolic pressure (LVEDP) were taken from the isolated heart preparations. Significant (P<0.05) differences are:

*vs. normoxia, Mixed Linear Model for birth weight, placental weight, fetal absolute and relative heart weights; ANOVA+Tukey Test for all other variables.

**Figure 2:**
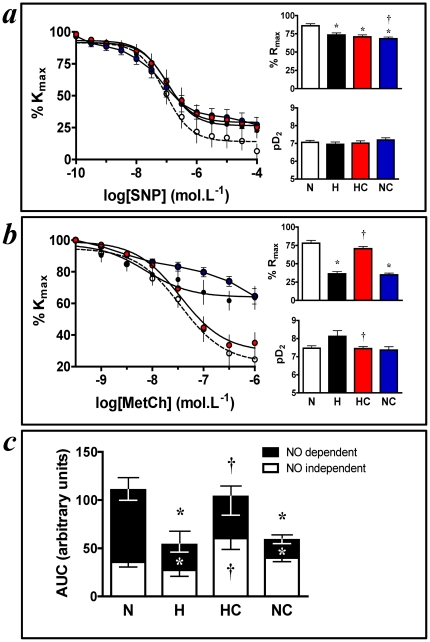
Femoral artery vasodilator function in adulthood. Values are mean±S.E.M. for the concentration-response curve (maximal response, %Rmax, and sensitivity, pD2) to sodium nitroprusside (SNP) *a* and to methacholine (MetCh) *b*, and for the nitric oxide (NO) dependent and independent components (area under the curve, AUC) of the endothelial dependent vasorelaxation *c* in femoral resistance arteries isolated from 4 month adult offspring. Concentration-response curves were analysed using an agonist-response best-fit line. The maximal relaxant response (%Rmax) was expressed as percentage of the contraction induced by PE and the vascular sensitivity was expressed as pD2 (-logEC50). The contribution of NO-dependent mechanisms to the relaxation induced by MetCh was calculated by subtracting the area under the curve (AUC) for MetCh – the AUC for MetCh + LNAME. The contribution of NO-independent mechanisms was calculated by the AUC for MetCh + LNAME [31]. Groups are: Normoxia, n = 8 (N, white symbols), Hypoxia, n = 8 (H, black symbols), Hypoxia+Vitamin C, n = 6 (HC, red symbols) and Normoxic+Vitamin C, n = 8 (NC, blue symbols). Significant (P<0.05) differences are: * vs. N, † vs. H, One-Way ANOVA with Tukey Test.

In adult offspring at 4 months of age, the endothelium-independent and endothelium-dependent relaxation in femoral resistance arteries was evaluated by generating cumulative concentration-response curves to the NO-donor sodium nitroprusside and to the acetylcholine mimetic methacholine, respectively. To determine the partial contributions of NO-dependent and NO-independent mechanisms to the endothelial dysfunction, additional concentration-response curves were determined following incubation with the NO synthase blocker L-NAME alone or after both L-NAME and the prostaglandin synthase inhibitor indomethacin. Femoral resistance arteries of adult offspring from hypoxic pregnancies showed diminished dilatation to nitroprusside and markedly impaired relaxation to methacholine via NO-dependent mechanisms (Fig. 2A–C). Maternal treatment with vitamin C restored the relaxant response to methacholine, but not to sodium nitroprusside, in femoral resistance arteries of adult offspring from hypoxic pregnancies via increasing the contribution of NO-independent mechanisms (Fig. 2A–C). Intriguingly, femoral resistance arteries of adult offspring from normoxic pregnancies treated with vitamin C also showed markedly impaired relaxation to methacholine via NO-dependent mechanisms (Fig. 2A–C).

**Figure 3:**
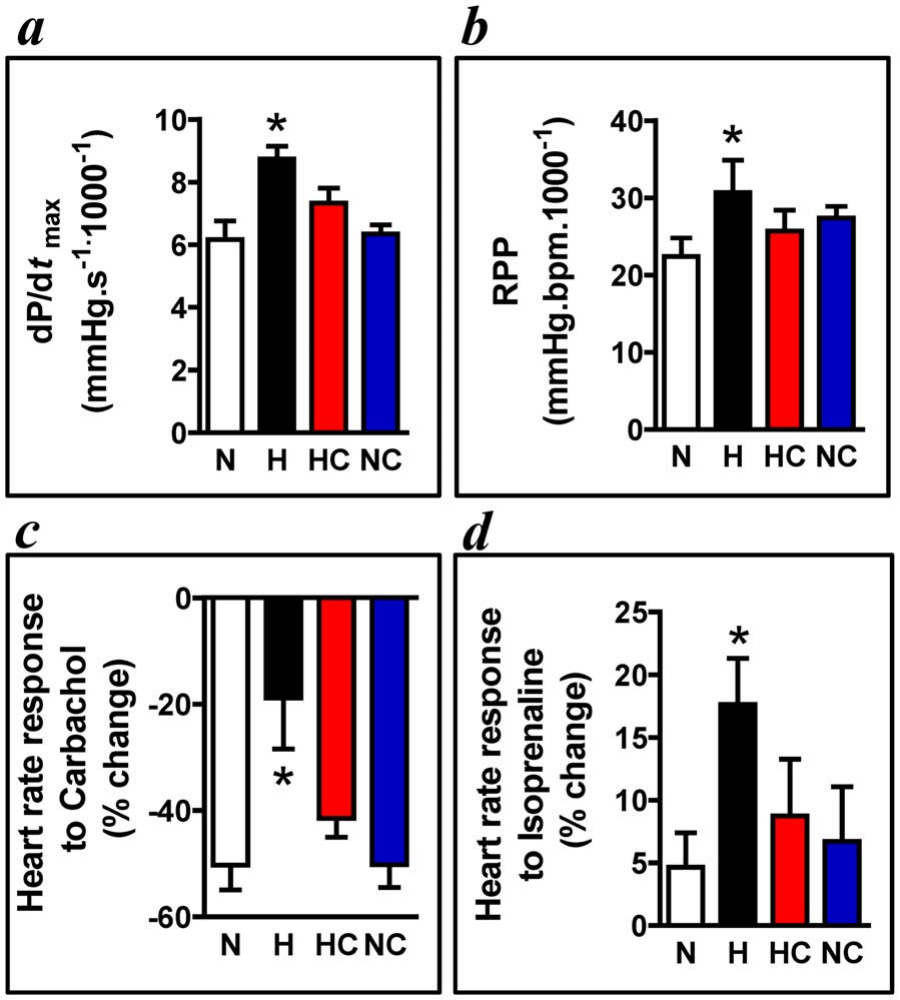
Cardiac function in adulthood. Values are mean±S.E.M. for the dP/dt max *a*, the heart rate-pressure product (RPP) *b*; and the heart rate responses to 10–6 M Carbachol *c* and to 10–7 M Isoprenaline *d*. Groups are: Normoxia, n = 6 (N,□), Hypoxia, n = 6 (H, ▪), Hypoxia+Vitamin C, n = 7 (HC, red histogram) and Normoxic+Vitamin C, n = 7 (NC, blue histogram). Significant (P<0.05) differences are: * vs. all, One-Way ANOVA with Tukey Test.

In adult offspring at 4 months of age, the body and cardiac weights were similar across all four groups studied. Neither the morphology of the heart or aorta or the expression levels of cardiac HSP70 were affected by hypoxic pregnancy or treatment of the pregnancy with vitamin C (Table 1). However, isolated hearts from adult offspring of hypoxic pregnancy showed significantly enhanced values for dP/dt_max_ and for the rate-pressure product (Fig. 3A and 3B). While the chronotropic response to the muscarinic agonist carbachol was significantly suppressed, this was markedly enhanced to the β_1_-adrenoreceptor agonist isoprenaline (Fig. 3C and 3D). Maternal treatment with vitamin C in hypoxic pregnancy restored these cardiac responses towards values measured in adult offspring from normoxic pregnancy. Vitamin C in normoxic pregnancy did not have any effect on cardiac responses in adult offspring (Fig. 3A–D). Basal heart rate, left ventricular developed pressure and left ventricular end-diastolic pressure in hearts of adult offspring were unaffected by hypoxic pregnancy or pregnancy treated with vitamin C (Table 1).

Maternal plasma levels of ascorbic acid were increased by similar extents at day 20 of gestation in normoxic and hypoxic pregnancy following maternal vitamin C (Table 1). Maternal and fetal haematocrit increased by similar extents in hypoxic pregnancy whether untreated or treated with vitamin C (Table 1). In normoxic pregnancy, maternal food intake did not vary significantly during gestation and averaged 29.4±0.7 g per day. Neither hypoxic pregnancy nor maternal treatment with vitamin C affected daily maternal food intake (H: 26.6±0.5 g; HC: 27.6±1.0 g; NC: 28.1±0.7 g.d^−1^) or litter size (Table 1). However, birth weight was significantly enhanced in both normoxic and hypoxic pregnancy following maternal treatment with vitamin C (Table 1). Hypoxic pregnancy with or without vitamin C increased placental weight (Table 1). Placental efficiency, calculated as the ratio of fetal body weight to placental weight at day 20 of gestation, revealed that hypoxic pregnancies had slightly lower calculated values for placental efficiency relative to controls (H = 5.59±0.11, n = 49 vs. C = 5.65±0.16, n = 38; P<0.05). Maternal treatment with vitamin C did not affect placental efficiency in normoxic or hypoxic pregnancy (NC = 5.87±0.09, n = 60; HC = 5.17±0.09, n = 60).

## Discussion

The data show that chronic prenatal hypoxia, leading to a significant increase in fetal haematocrit, promotes fetal aortic wall thickening and oxidative stress in the fetal heart and vasculature by the end of gestation. By adulthood, these effects resolve but prenatal chronic hypoxia sets a functional deficit in both the heart and the peripheral circulation. Adult offspring from chronically hypoxic pregnancies show enhanced myocardial contractility due to sympathetic dominance and NO-dependent endothelial dysfunction in peripheral resistance vessels. The effects of chronic prenatal hypoxia on the fetal and adult offspring cardiovascular system are prevented by maternal treatment with vitamin C during pregnancy. While it is established that chronic prenatal hypoxia programmes cardiovascular disease, the mechanisms mediating this programming had remained elusive. This has prevented the identification of potential therapeutic targets for clinical intervention. Therefore, the primary novelty of the discoveries reported here is that programming by prenatal chronic hypoxia of cardiac and vascular dysfunction in adulthood follows the induction of oxidative stress in the fetal heart and vasculature, and that cardiac and endothelial dysfunction in adulthood can both be prevented by maternal treatment with antioxidants during pregnancy. Therefore, the study supports the hypothesis tested, and the new discoveries provide insight to mechanism and intervention using a broad range of measurements at several levels in a single longitudinal experiment.

### Effects on the vasculature

Several studies have reported that chronic hypoxia during development promotes a vasoconstrictor phenotype in peripheral resistance circulations of fetal offspring. Chronic hypoxia may achieve this by increasing sympathetic innervation [12], [32] and the responsiveness of peripheral circulations to α_1_-adrenergic agonists [33] in the fetus. In addition, chronic hypoxia may reduce fetal vasodilator capacity by affecting endothelial function. For instance, the interaction between O_2_^•−^: NO promotes a vascular oxidant ratio that is also an important determinant of vascular tone [34], and we have shown that hypoxia can manipulate this vascular oxidant ratio in fetal resistance circulations towards vasoconstriction [35]. Two studies have further reported that this vasoconstrictor phenotype triggered by developmental hypoxia is not only present in fetal life, but that chronic prenatal hypoxia can programme permanent endothelial dysfunction in resistance circulations of the adult offspring [25], [26]. Here, we show that maternal treatment with antioxidants during pregnancy can restore this programmed impaired vasodilator phenotype in adult offspring, providing new evidence for the mechanism mediating the developmental programming of endothelial dysfunction by prenatal chronic hypoxia to be due to vascular oxidative stress.

It is interesting that the balance of redox modulation of vascular tone, imposed by the O_2_^•−^: NO ratio, may be tipped in either direction to promote disequilibrium, as maternal treatment with vitamin C in normoxic pregnancy also promoted endothelial dysfunction. Maternal antioxidant supplementation may therefore only restore the offspring vascular dysfunction in pregnancy conditions associated with increased O_2_^•−^ generation and vascular oxidative stress. Conversely, antioxidant treatment in healthy conditions where the offspring vascular physiology is already replenished with an appropriate redox balance may, in fact, lead to excess NO bioavailability, tipping the balance in the opposite direction. Excess NO bioavailability is known to promote peroxynitrite generation, thereby triggering mechanistic side-effects resembling those of vascular oxidative stress [27]. The implications of these data are that maternal treatment with antioxidants may provide possible therapy against the programming effects on vascular dysfunction in pregnancy complicated by fetal hypoxia, such as during placental insufficiency, preeclampsia, gestational diabetes or high altitude pregnancy. However, the data underline that excessive antioxidant supplementation in healthy pregnancy may, in effect, be detrimental and is not to be recommended.

Experimental studies have also reported that chronic prenatal hypoxia promotes thickening of the aortic wall in the fetal offspring [12]–[15]. At least five independent clinical studies have reported that babies from pregnancies complicated by placental insufficiency show aortic wall thickening [36]–[40]. This is particularly relevant in the clinical setting, as thickening of the aortic wall has been hailed as a key component in the aetiology of coronary heart disease [41] and it is also as the first physical sign in the development of atherosclerosis [41]. Further, measurement of aortic pulse-wave velocity rather than of systolic blood pressure is recognised to be a better indicator of later cardiovascular disease, including impaired coronary artery flow and left ventricular dysfunction [42]. The peripheral vasoconstrictor phenotype and aortic wall thickening in offspring of hypoxic pregnancies in the present study may therefore be linked, as there is general agreement that vascular remodelling of this type can result from an increase in cardiac afterload [15], [43]. Accordingly, maternal treatment with antioxidants in hypoxic pregnancy restored aortic wall thickening and the endothelial dysfunction in resistance circulations in the offspring.

### Effects on the heart

A number of comprehensive studies have also shown that developmental hypoxia can programme cardiac dysfunction in adult offspring. Adverse programmed effects include altered myocardial structure and metabolism, a decline in cardiac performance and heightened cardiac susceptibility to adult ischaemic injury [15]–[24]. These experimental studies are of substantial clinical relevance, as it has now also been reported that children from complicated pregnancies show changes in cardiac morphology and function [40]. Here, we show that chronic prenatal hypoxia has permanently altered the mechanical properties of the myocardium and its inherent response to chemical mediators of contractile force. An increase in dP/dt_max_ is an established index of increased myocardial contractility and an increase in the rate-pressure product is associated with increased myocardial work load and oxygen consumption [44]. The mechanism driving the enhanced myocardial contractility is enhanced responsiveness to β_1_-adrenoreceptor stimulation, coupled with decreased reactivity to muscarinic agonists. It is possible that the increase in myocardial contractility occurs in response to the increased afterload derived from the increased peripheral vascular impedance. We also show that the effects of chronic prenatal hypoxia on the heart of adult offspring can be prevented by maternal antioxidant treatment during pregnancy, providing new evidence for the mechanism driving the developmental programming of heart dysfunction by prenatal hypoxia to also be secondary to oxidative stress. Maternal treatment with antioxidants may prevent programmed autonomic influences on the adult heart triggered by prenatal hypoxia by altering the bioavailability of NO, as the gaseous neurotransmitter can enhance myocardial vagal dominance [45]. Sustained increases in myocardial contractility due to heightened sympathetic excitation and diminished parasympathetic reactivity are strongly associated with cardiovascular disease, and this cardiac phenotype is a known predictor of eventual heart failure in humans [45], [46].

### Dose of vitamin C

In vascular endothelial cells, nitric oxide (NO) is produced constitutively *in vivo*, but it is rapidly inactivated by superoxide anion (^•^O_2_^−^) to produce ^•^ONOO^−^ [47]. Although the availability of ^•^O_2_^−^ in tissues is strictly limited by the abundance of superoxide dismutase (SOD), which is able to dismutate ^•^O_2_^−^ at a rapid rate constant [48], NO can still compete effectively with SOD for ^•^O_2_^−^. Jackson and colleagues [49] reported that vitamin C could scavenge ^•^O_2_^−^ at low concentrations, but it could only prevent the impairment by ^•^O_2_^−^ of endothelium derived NO-mediated arterial relaxation at much higher physiological concentrations. Therefore, the capacity of vitamin C to scavenge ^•^O_2_^−^ and its ability to prevent the interaction between NO and ^•^O_2_^−^ appear to occur at very different concentrations *in vivo*. The dose of vitamin C used in the present study was derived from a previous study in our laboratory which achieved elevations in circulating ascorbate concentrations, enabling it to act as an antioxidant *in vivo* in ovine pregnancy [35], thereby justifying the antioxidant dosing regimen. In rat pregnancy, this equated to *ca*. 0.9 g kg^−1^·d^−1^ of vitamin C administration. Although this dose of vitamin C far exceeds that given to pregnant women, for instance in all reported clinical trials against preeclampsia (1 g per day per woman [50]–[54]), the increment from baseline in circulating ascorbate concentrations measured in dams in the present study was of *ca.* 70% and, therefore, similar to the increment achieved in pregnant women in the VIP trial following maternal vitamin C administration [50]. Since treatment of human patients with high doses of vitamin C can promote oxaluria and the risk of kidney stones [55], vitamin C may not be the antioxidant of choice for human therapy in complicated pregnancy. However, the study provides the proof of principle that maternal antioxidant treatment does protect against the programming of cardiovascular dysfunction in offspring of pregnancy complicated by fetal hypoxia.

### Maternal food intake and biometry

Previous studies have shown that prenatal hypoxia in the last third of gestation decreases maternal food intake and induces disproportionate fetal growth restriction [11], [13], [25]. In our study, developmental hypoxia throughout most of gestation did not affect maternal food intake or fetal growth, but it increased placental weight. These differences are important because they highlight that programming of cardiovascular dysfunction is due to prenatal hypoxia alone independent of maternal nutrition. Further, the model shows that alterations in fetal and/or postnatal growth are not a prerequisite for developmental programming. The differences in the placental and fetal phenotypes between the studies are likely due to the differential temporal growth demands of the placenta and fetus during pregnancy. In the rat, whereas placental growth starts early in gestation and continues throughout pregnancy, fetal growth is exponential and maximal by the end of gestation [56]. Therefore, early-onset hypoxia may stimulate greater than normal placental growth, cushioning the adverse effects of the challenge on fetal growth. Accordingly, studies of human pregnancy at high altitude have reported improved placental vascularisation with increased placental capillary diameter, capillary length and capillary volume [57], [58]. Further data reported in this study show that maternal treatment with vitamin C increased birth weight in both normoxic and hypoxia pregnancy. The mechanism mediating this may be secondary to the increased NO bioavailbility enhancing placental perfusion, as we have previously also reported that maternal treatment with antioxidants can enhance umbilical blood flow and fetal growth via NO-dependent mechanisms [59], [60].

### Clinical perspective

A meta-analysis of randomised clinical trials on the effectiveness of antioxidants in cardiovascular risk reduction has shown invariably negative results [61]. Without exception, all of these studies have been on patients with established cardiovascular disease, providing evidence that antioxidant treatment is unlikely to rectify cardiovascular function once disease is established. Our studies provide a different approach, addressing the effects of antioxidant therapy in the fetus as a preventive strategy, halting the slow development of cardiovascular dysfunction across the life span at its very origin. Therefore, the data offer insight into mechanism and possible targets for clinical intervention against the developmental programming of heart and peripheral vascular dysfunction in risky pregnancy.

## References

[1] Basson M (2008). Cardiovascular Disease.. Nature.

[2] Agarwal A, Williams GH, Fisher NDL (2005). Genetics of human hypertension.. Trends Endocrin Metab (Review).

[3] Barker DJP (1998). Mothers, Babies, and Disease in Later Life..

[4] Gluckman PD, Hanson MA, Cooper C, Thornburg KL (2008). Effect of in utero and early-life conditions on adult health and disease.. NEJM.

[5] Lucas A (1991). Programming by early nutrition in man.. Ciba Found Symp.

[6] McMillen IC, Robinson JS (2005). Developmental origins of the metabolic syndrome: prediction, plasticity, and programming.. Physiol Rev.

[7] Armitage JA, Taylor PD, Poston L (2005). Experimental models of developmental programming: consequences of exposure to an energy rich diet during development.. J Physiol.

[8] Giussani DA, Spencer JA, Moore PJ, Bennet L, Hanson MA (1993). Afferent and efferent components of the cardiovascular reflex responses to acute hypoxia in term fetal sheep.. J Physiol.

[9] Giussani DA, Gluckman PD, Hanson MA (2006). Prenatal hypoxia: Relevance to developmental origins of health and disease.. Developmental Origins of Health and Disease.

[10] Richardson BS, Bocking AD (1998). Metabolic and circulatory adaptations to chronic hypoxia in the fetus.. Comp Biochem Physiol A Mol Integ Physiol.

[11] Morrison JL (2008). Sheep models of intrauterine growth restriction: fetal adaptations and consequences. Review.. Clin Exp Pharm Physiol.

[12] Rouwet EV, Tintu AN, Schellings MW, van Bilsen M, Lutgens E (2002). Hypoxia induces aortic hypertrophic growth, left ventricular dysfunction, and sympathetic hyperinnervation of peripheral arteries in the chick embryo.. Circulation.

[13] Camm EJ, Hansell JA, Kane AD, Herrera EA, Lewis C (2010). Partial contributions of developmental hypoxia and undernutrition to prenatal alterations in somatic growth and cardiovascular structure and function.. Am J Obstet Gynecol.

[14] Salinas CE, Blanco CE, Villena M, Camm EJ, Tuckett JD (2010). Developmental origin of cardiac and vascular disease in chick embryos incubated at high altitude.. JDOHaD.

[15] Tintu A, Rouwet E, Verlohren S, Brinkmann J, Ahmad S (2009). Hypoxia induces dilated cardiomyopathy in the chick embryo: mechanism, intervention, and long-term consequences.. PLoS One.

[16] Ream M, Ray AM, Chandra R, Chikaraishi DM (2008). Early fetal hypoxia leads to growth restriction and myocardial thinning.. Am J Physiol Regul Integr Comp Physiol.

[17] Li G, Xiao Y, Estrella JL, Ducsay CA, Gilbert RD (2003). Effect of fetal hypoxia on heart susceptibility to ischemia and reperfusion injury in the adult rat.. J Soc Gynecol Invest.

[18] Zhang L (2005). Prenatal hypoxia and cardiac programming.. J Soc Gynecol Investig.

[19] Xue Q, Zhang L (2009). Prenatal hypoxia causes a sex-dependent increase in heart susceptibility to ischemia and reperfusion injury in adult male offspring: role of protein kinase C epsilon.. J Pharmacol Exp Ther.

[20] Patterson AJ, Chen M, Xue Q, Xiao D, Zhang L (2010). Chronic prenatal hypoxia induces epigenetic programming of PKC{epsilon} gene repression in rat hearts.. Circ Res.

[21] Patterson AJ, Zhang L (2010). Hypoxia and fetal heart development.. Curr Mol Med.

[22] Xue Q, Dasgupta C, Chen M, Zhang L (2011). Foetal hypoxia increases cardiac AT(2)R expression and subsequent vulnerability to adult ischaemic injury.. Cardiovasc Res.

[23] Rueda-Clausen CF, Morton JS, Lopaschuk GD, Davidge ST (2011). Long-term effects of intrauterine growth restriction on cardiac metabolism and susceptibility to ischaemia/reperfusion.. Cardiov Res.

[24] Hauton D, Ousley V (2009). Prenatal hypoxia induces increased cardiac contractility on a background of decreased capillary density.. BMC Cardiovasc Disord.

[25] Williams SJ, Hemmings DG, Mitchell JM, McMillen IC, Davidge ST (2005). Effects of maternal hypoxia or nutrient restriction during pregnancy on endothelial function in adult male rat offspring.. J Physiol.

[26] Morton JS, Rueda-Clausen CF, Davidge ST (2011). Flow-mediated vasodilation is impaired in adult rat offspring exposed to prenatal hypoxia.. J Appl Physiol.

[27] Halliwell B, Gutteridge JMC (2004). Free Radicals in Biology and Medicine.. Oxford University Press.

[28] Herrera EA, Camm EJ, Cross CM, Mullender JL, Wooding FB (2011). Morphological and Functional Alterations in the Aorta of the Chronically Hypoxic Fetal Rat.. J Vasc Res.

[29] Iriyama K, Teranishi T, Mori H, Nishiwaki H, Kusaka N (1984). Simultaneous determination of uric and ascorbic acids in human serum by reversed-phase high-performance liquid chromatography with electrochemical detection.. Anal. Biochem.

[30] Pagano G, Degan P, d'Ischia M, Kelly FJ, Pallardó FV (2004). Gender- and age-related distinctions for the in vivo prooxidant state in Fanconi anaemia patients.. Carcinogenesis.

[31] Herrera EA, Verkerk MM, Derks JB, Giussani DA (2010). Antioxidant treatment alters peripheral vascular dysfunction induced by postnatal glucocorticoid therapy in rats.. PLoS One.

[32] Ruijtenbeek K, le Noble FA, Janssen GM, Kessels CG, Fazzi GE (2000). Chronic hypoxia stimulates periarterial sympathetic nerve development in chicken embryo.. Circulation.

[33] Kim YH, Veille JC, Cho MK, Kang MS, Kim CH (2005). Chronic hypoxia alters vasoconstrictive responses of femoral artery in the fetal sheep.. J Korean Med Sci.

[34] Chen CA, Wang TY, Varadharaj S, Reyes LA, Hemann C (2010). S-glutathionylation uncouples eNOS and regulates its cellular and vascular function.. Nature.

[35] Thakor AS, Richter HG, Kane AD, Dunster C, Kelly FJ (2010). Redox modulation of the fetal cardiovascular defence to hypoxaemia.. J Physiol.

[36] Skilton MR, Evans N, Griffiths KA, Harmer JA, Celermajer DS (2005). Aortic wall thickness in newborns with intrauterine growth restriction.. Lancet.

[37] Koklu E, Kurtoglu S, Akcakus M, Koklu S, Buyukkayhan D (2006). Increased aortic intima-media thickness is related to lipid profile in newborns with intrauterine growth restriction.. Horm Res.

[38] Akira M, Yoshiyuki S (2006). Placental circulation, fetal growth, and stiffness of the abdominal aorta in newborn infants.. J Pediatr.

[39] Cosmi E, Visentin S, Fanelli T, Mautone AJ, Zanardo V (2009). Aortic intima media thickness in fetuses and children with intrauterine growth restriction.. Obstet Gynecol.

[40] Crispi F, Bijnens B, Figueras F, Bartrons J, Eixarch E (2010). Fetal growth restriction results in remodeled and less efficient hearts in children.. Circulation.

[41] Rader DJ, Daugherty A (2008). Translating molecular discoveries into new therapies for atherosclerosis.. Nature.

[42] Cruickshank K, Riste L, Anderson SG, Wright JS, Dunn G (2002). Aortic pulse-wave velocity and its relationship to mortality in diabetes and glucose intolerance: an integrated index of vascular function?. Circulation.

[43] Kitanaka T, Alonso JG, Gilbert RD, Siu BL, Clemons GK (1989). Fetal responses to long-term hypoxemia in sheep.. Am J Physiol.

[44] Gobel FL, Norstrom LA, Nelson RR, Jorgensen CR, Wang Y (1978). The rate-pressure product as an index of myocardial oxygen consumption during exercise in patients with angina pectoris.. Circulation.

[45] Danson EJ, Li D, Wang L, Dawson TA, Paterson DJ (2009). Targeting cardiac sympatho-vagal imbalance using gene transfer of nitric oxide synthase.. J Mol Cel Cardiol.

[46] Bristow MR (2002). Beta-adrenergic receptor blockade in chronic heart failure.. Circulation.

[47] Kissner R, Nauser T, Bugnon P, Lye PG, Koppenol WH (1997). Formation and properties of peroxynitrite as studied by laser flash photolysis, high-pressure stopped-flow technique, and pulse radiolysis volume.. Chem. Res. Toxicol.

[48] Fridovich I (1978). Oxygen radicals, hydrogen peroxide and oxygen toxicity..

[49] Jackson TS, Xu A, Vita JA, Keaney JF (1998). Ascorbate prevents the interaction of superoxide and nitric oxide only at very high physiological concentrations.. Circ Res.

[50] Poston L, Briley AL, Seed PT, Kelly FJ, Shennan AH (2006). Vitamins in Pre-eclampsia (VIP) Trial Consortium (2006) Vitamin C and vitamin E in pregnant women at risk for pre-eclampsia (VIP trial): randomised placebo-controlled trial.. Lancet.

[51] Spinnato JA, Freire S, Pinto E, Silva JL, Cunha Rudge MV (2007). Antioxidant therapy to prevent preeclampsia: a randomized controlled trial.. Obstet Gynecol.

[52] Kontic-Vucinic O, Terzic M, Radunovic N (2008). The role of antioxidant vitamins in hypertensive disorders of pregnancy.. J Perinat Med.

[53] Rumbold A, Duley L, Crowther CA, Haslam RR (2008). Antioxidants for preventing pre-eclampsia.. Cochrane Database Syst Rev.

[54] Villar J, Purwar M, Merialdi M, Zavaleta N, Thi Nhu Ngoc N (2009). World Health Organisation multicentre randomised trial of supplementation with vitamins C and E among pregnant women at high risk for pre-eclampsia in populations of low nutritional status from developing countries.. BJOG.

[55] Massey LK, Liebman M, Kynast-Gales SA (2005). Ascorbate increases human oxaluria and kidney stone risk.. J Nutr.

[56] Witlin AG, Li ZY, Wimalawansa SJ, Grady JJ, Grafe MR (2002). Placental and fetal growth and development in late rat gestation is dependent on adrenomedullin.. Biol Reprod.

[57] Mayhew TM (2003). Changes in fetal capillaries during preplacental hypoxia: growth, shape remodelling and villous capillarization in placentae from high-altitude pregnancies.. Placenta.

[58] Cartwright JE, Keogh RJ, Tissot van Patot MC (2007). Hypoxia and placental remodelling.. Adv Exp Med Biol.

[59] Thakor AS, Herrera EA, Serón-Ferré M, Giussani DA (2010). Melatonin and vitamin C increase umbilical blood flow via nitric oxide-dependent mechanisms.. J Pineal Res.

[60] Richter HG, Hansell JA, Raut S, Giussani DA (2009). Melatonin improves placental efficiency and birth weight and increases the placental expression of antioxidant enzymes in undernourished pregnancy.. J Pineal Res.

[61] Kris-Etherton PM, Lichtenstein AH, Howard BV, Steinberg D, Witztum JL (2004). Nutrition Committee of the American Heart Association Council on Nutrition, Physical Activity, and Metabolism (2004) Antioxidant vitamin supplements and cardiovascular disease.. Circulation.

